# Imaging Modalities in Inflammatory Breast Cancer (IBC) Diagnosis: A Computer-Aided Diagnosis System Using Bilateral Mammography Images

**DOI:** 10.3390/s23010064

**Published:** 2022-12-21

**Authors:** Buket D. Barkana, Ahmed El-Sayed, Rana H. Khaled, Maha Helal, Hussein Khaled, Ruba Deeb, Mark Pitcher, Ruth Pfeiffer, Marilyn Roubidoux, Catherine Schairer, Amr S. Soliman

**Affiliations:** 1 Department of Electrical Engineering, University of Bridgeport, Bridgeport, CT 06604, USA; 2 National Institute of Cancer, Cairo University, Cairo 11796, Egypt; 3 Bioengineering Department, University of Bridgeport, Bridgeport, CT 06604, USA; 4 College of Health Sciences, University of Bridgeport, Bridgeport, CT 06604, USA; 5 Biostatistics Branch, National Cancer Institute, National Institute of Health (NIH), Bethesda, MD 20892, USA; 6 Department of Radiology, University of Michigan, Ann Arbor, MI 48109, USA; 7 Independent Researcher, Bethesda, MD 20892, USA; 8 City University of New York Medical School, New York, NY 10031, USA

**Keywords:** inflammatory breast cancer (IBC), medical imaging, mammography, CADx system, fuzzy logic

## Abstract

Inflammatory breast cancer (IBC) is an aggressive type of breast cancer. It leads to a significantly shorter survival than other types of breast cancer in the U.S. The American Joint Committee on Cancer (AJCC) defines the diagnosis based on specific criteria. However, the clinical presentation of IBC in North Africa (Egypt, Morocco, and Tunisia) does not agree, in many cases, with the AJCC criteria. Healthcare providers with expertise in IBC diagnosis are limited because of the rare nature of the disease. This paper reviewed current imaging modalities for IBC diagnosis and proposed a computer-aided diagnosis system using bilateral mammograms for early and improved diagnosis. The National Institute of Cancer in Egypt provided the image dataset consisting of IBC and non-IBC cancer cases. Type 1 and Type 2 fuzzy logic classifiers use the IBC markers that the expert team identified and extracted carefully. As this research is a pioneering work in its field, we focused on breast skin thickening, its percentage, the level of nipple retraction, bilateral breast density asymmetry, and the ratio of the breast density of both breasts in bilateral digital mammogram images. Granulomatous mastitis cases are not included in the dataset. The system’s performance is evaluated according to the accuracy, recall, precision, F1 score, and area under the curve. The system achieved accuracy in the range of 92.3–100%.

## 1. Introduction

Inflammatory breast cancer (IBC) is a rare and aggressive clinicopathologic form of breast cancer. It has a roughly 55% 5-year survival rate [1,2], much lower than most other types of breast cancer, which is about a 90% 5-year survival rate in the United States [3,4]. IBC rates are much higher in North Africa than in the United States, ranging from 5 to 7% in Morocco and Tunisia [5,6] to about 11% in Egypt [7], compared to 2–4% in the United States. 

The American Joint Committee on Cancer (AJCC) defines the IBC with erythema (redness), edema, and peau d’orange over at least a third of the breast—often with no underlying tumor mass—with a duration of the first symptom to the diagnosis fewer than six months. In addition, the histopathology of tumor emboli in the breast biopsy indicates IBC. However, the diagnosis of IBC based on the AJCC criteria is challenging because the presence and extent of erythema, edema, and peau d’orange vary between IBC patients, and there are no solid pathologic criteria to confirm the diagnosis of the diseases [8,9]. In addition, the AJCC clinical signs are not present in many IBC cases. Many IBC patients have symptoms that are different from the restrictive AJCC criteria. They may have no visible redness, less than one-third of the breast, or redness, but no peau d’orange or edema [8,10]. Furthermore, the clinical diagnosis of IBC based on redness, peau d’orange, and edema may be further hampered for women with dark skin and large breasts [11]. The epidemiologic and clinical research in Egypt, Tunisia, and Morocco have shown variations in the presence and extent of erythema, edema, and peau d’orange in clinically confirmed IBC patients [7,9,12,13]. 

**Figure 1:**
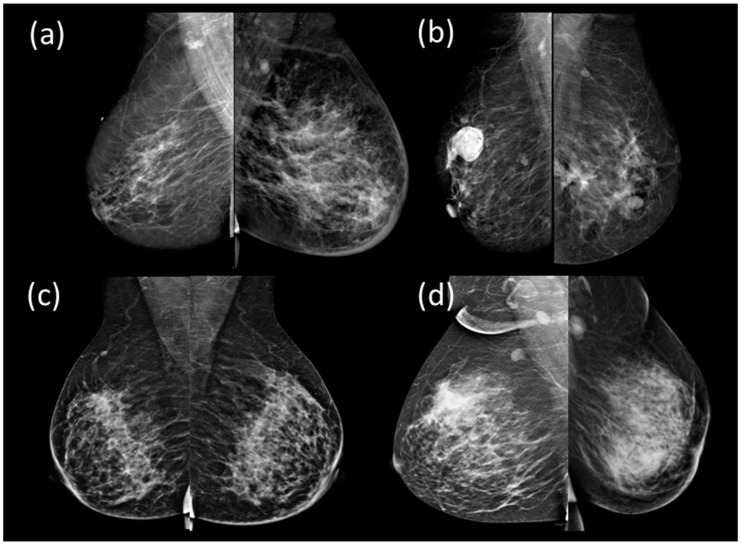
MLO views of four digital bilateral mammography images showing (**a**) IBC (present at the left breast), (**b**) non-IBC cancer, (**c**) benign, and (**d**) chronic granulomatous mastitis (CGM) (present at the left breast).

Routine screening and imaging play an essential role in the characterization of a tumor for image-guided biopsy, the definition of locoregional disease, the diagnosis of distant metastases, and the evaluation of response to therapy [14,15]. Advances in medical imaging have improved the diagnosis and staging of IBC. However, analyzing the imaging modalities and diagnosing IBC requires expertise, which is limited, especially in low-income countries. Sonography is helpful as a localizing tool for biopsy patients with identifiable masses and for evaluating the regional nodes. Furthermore, sonography shows primary breast lesions more frequently visible than mammography [16]. Magnetic resonance imaging (MRI) also may help guide skin punch biopsies because of its high sensitivity for demonstrating parenchymal breast lesions, skin thickening, and enhancement. Mammography is one of the primary imaging modalities used in IBC diagnosis worldwide. Mammography shows palpable tumor mass, a large calcification area, and/or parenchymal distortion, and skin thickening over the breast with or without a breast mass [16]. Full-field digital mammography continues to be one of the imaging lines of IBC diagnosis as it assesses breast parenchyma in patients suspected of IBC. In the initial stages, skin thickening was mainly seen in the inferior areolar region [17]. Stromal coarsening or trabecular thickening and diffuse increase of parenchymal density/diffuse asymmetry constitute the main mammographic findings of IBC [18]. Furthermore, Gunhan-Bilgen reported that intramammary mass was found in 16% of cases, architectural distortion is rare (approximately 56% of cases), asymmetric focal density was found in 61% of cases, and micro-calcification are less commonly seen in mammography in 56% of cases [16,17,18]. A linear relationship was also reported between increased background breast parenchyma density (BIRADS 3 and 4) and IBC [16]. Axillary lymphadenopathy and breast masses may also be detected on mammography, but they are best evaluated with ultrasonography [19]. Figure 1 shows the mediolateral oblique (MLO) views of bilateral digital mammograms for IBC, non-IBC cancer cases, benign, and chronic granulomatous mastitis (CGM) cases. Figure 1a shows a biopsy-proven IBC case on the left breast. Skin thickening, nipple retraction, and breast density exist on the left breast image. Figure 1b shows non-IBC cancer, which does not show any skin thickening. Figure 1c represents a benign case and Figure 1d shows a biopsy-proven CGM on the left breast. There are abnormal size lymph nodes in the axilla due to the infection. There is no skin thickening on the right breast and the axillary nodes are normal.

With the advances in imaging, reported analysis, and findings in the literature, this paper proposes a model to extract IBC markers and a rule-based fuzzy logic diagnosis model using mammography images. To our knowledge, there is no reported computer-aided diagnosis (CADx) model to diagnose IBC from any imaging modalities. Our work will provide insights into possible CADx systems for IBC detection using image processing methodologies and machine learning algorithms. This work aims to answer the following questions: (1) Which IBC symptoms can be detected using image processing methods from mammography images? (2) What is the achievability of CAD systems for IBC? 

Understanding the effectiveness of image processing methods and computer-aided designs will advance the early diagnosis of this fatal disease. Moreover, it might influence the field to establish a public-domain IBC imaging database, enabling interdisciplinary researchers to develop detection and diagnosis systems. Since IBC is so rare, most imaging datasets are small and not publicly available to the research community. The main contributions of this work are:

(1) Developing segmentation and measurement algorithms for breast density and breast skin thickness.
(2) Extracting markers of IBC in digital mammogram images.
(3) Designing a fuzzy logic CADx system for early IBC detection.
(4) Comparing the performance of two common rule-based techniques for IBC diagnosis.
(5) Reporting challenges in the process of CAD for IBC diagnosis.
(6) Influence establishing a public-domain IBC imaging database.

The paper is organized as follows: Section 2 reviews the imaging modalities in inflammatory breast cancer diagnosis. The methodology is presented in Section 3. Type-1 and Type-2 fuzzy logic CAD models are designed in Section 4, and the feasibility and effectiveness of ML-based IBC diagnosis systems are analyzed and discussed in Section 5. 

## 2. Review of Imaging Modalities in Inflammatory Breast Cancer Diagnosis

Several studies have been published between 2000 and 2022 regarding imaging modalities, image processing and analysis, and computer-aided diagnosis algorithms through PubMed, Google Scholar, and Science Direct using the keywords: “inflammatory breast cancer”, “breast cancer”, “radiology”, and “imaging modalities”. There is limited literature on IBC findings in imaging modalities. However, those findings carry some similarities and differences between studies. Here, we mention some of the significant works that motivated the proposed CAD model in our work. 

Early work in 2005 studied the IBC characteristics of nine patients using mammography, ultrasonography, and MRI. IBC showed skin thickening and nipple-areolar swelling on mammography, ultrasonography, and MRI. It was reported that MRI enhancement of thickened skin and parenchyma could be valuable findings in diagnosing inflammatory breast cancer [20]. Another early study investigated the IBC findings in mammography and sonography and discussed the registration and fusion between parametric MRI and F-fluorodeoxyglucose (FDG) positron emission tomography/computed tomography (PET/CT) (FDG-PET/CT) imaging modalities on the same patients. It was reported that image registration and fusion might help detect IBC [21].

Renz et al. compared the MRI findings of 48 patients with IBC and 52 patients with non-inflammatory locally advanced breast carcinoma (LABC). Both groups were similar regarding age and histopathologic subtypes. The study found no significant differences between groups regarding the dynamic tumor signal characteristics, prominent vessels, perifocal edema, axillary lymph node involvement, the morphology of focal masses, and morphologic pattern of non-mass-like enhancement. Furthermore, the number of focal masses and the spatial distribution of the tumoral infiltration significantly differed between IBC and non-IBC cases in MRI [22].

Yang et al. carried out work for eighty patients (ranging from 25 to 78 years old; the median age was 51) using mammography, sonography, MRI, and PET/CT. All patients presented with breast erythema, skin edema, peau d’orange changes, and other clinical findings, including palpable breast masses, swelling, firmness, and nipple retraction. Only 19% of the patients had no associated clinical results besides erythema. The percentage of patients who had undergone mammography, sonography, MRI, and PET/CT were 94%, 95%, 41%, and 30%, respectively. A primary breast parenchymal lesion (BPL) was found in 80% of the patients on mammography (mass or calcifications), 95% on sonography (mass or architectural distortion and skin thickening), 100% on MRI (malignant enhancing BPL), and 96% on PET/CT (hypermetabolic BPL). MRI showed skin thickening and enhancement in 94% of patients. The work reported that MRI was the most accurate imaging technique in detecting a primary BPL in IBC patients, while sonography can help diagnose regional nodal disease. The study suggested MRI as the preferred initial imaging modality for IBC and PET/CT as a companion for detecting distant metastases [16].

IBC markers in MRI, mammography, and ultrasound images of eighty patients with a clinical diagnosis of IBC were studied to define the role of MRI in IBC diagnosis [23]. The study showed that a primary breast lesion was detected in 98%, 68%, and 94% of the patients by MRI, mammography, and ultrasound, respectively. In MRI and mammography, skin thickening was seen in 93% and 72% of the patients. Vertakova-Krakovska and Vanovcanova reported that 31 IBC patients underwent pre-treatment core-cut, punch skin biopsy, and MRI in response to neoadjuvant chemotherapy (NAC) (doxorubicin and/or taxane regimen, HER2+ with trastuzumab if applicable). MRI assessed patients after a second cycle and confirmed all cases of IBC correctly. The study reported an initial MRI as a superior and reliable method for diagnosing IBC [24].

Another study comparing mammography, ultrasonography, and MRI was carried out by Alunni in 2021. The work reported that (1) mammography shows skin thickening, trabecular thickening, and an overall increase in the density of the breast; (2) mammography is the least sensitive technique for detecting intramammary masses in an inflammatory context; (3) ultrasonography shows the thickening of the skin and an increase in echogenicity of the breast parenchyma; (4) ultrasound can be used to detect breast masses and search for multifocality with greater sensitivity than mammography; (5) the role of MRI in IBC diagnosis is much debated both for pretherapeutic assessment and for monitoring during chemotherapy; (6) the differential diagnosis with infectious mastitis may also be difficult [18].

Most studies reported that MRI is the most accurate imaging modality in diagnosing IBC. However, MRI may not be easily accessible to women in low and middle-income countries (LMIC) and underserved areas. Bilateral mammograms followed by bilateral breast and ultrasounds are considered the initial imaging modalities because of their low cost and easy accessibility [8,17]. Mammographic findings in IBC patients include skin thickening (84–93%), trabecular thickening (62–81%), trabecular distortion (37%), increased breast density (93%), calcifications (47–56%), and axillary adenopathy (24%) [8]. Studies showed that about half of the IBC cases had mammographic evidence of nipple inversion [8,17]. While Mamouch et al. reported that 25% of IBC cases do not show tumor mass [25], another study reported breast mass is visible in only 15% of patients [26]. 

## 3. Methodology

Given what the literature reported, we used the most common mammographic findings—skin thickening, and density features—in this proposed computer-aided diagnosis (CAD) model. It consists of preprocessing the bilateral digital mammography images, segmentation, feature extraction, and fuzzy logic stages. The steps of the CAD are explained in the following subsections.

### 3.1. Dataset

The dataset is a collection of six IBC and eight non-IBC breast cancer digital mammography images, including the craniocaudal (CC) and MLO views collected by the National Institute of Cancer, Cairo University, in Egypt, where the patient’s consent was obtained. They are all high resolution with an average of 2355 × 1315 pixels. GE Healthcare Senographe DS Mammography System was used. Data were gathered and stored in a DICOM format. Some irrelevant annotations were removed, such as the patient’s name, identification, date of the study, and the image series. The Digital Imaging and Communications in Medicine (DICOM) images were then exported losslessly to a joint photographic experts group format using the RadiAnt DICOM viewer application [27].

### 3.2. Preprocessing and Segmentation

**Figure 2:**
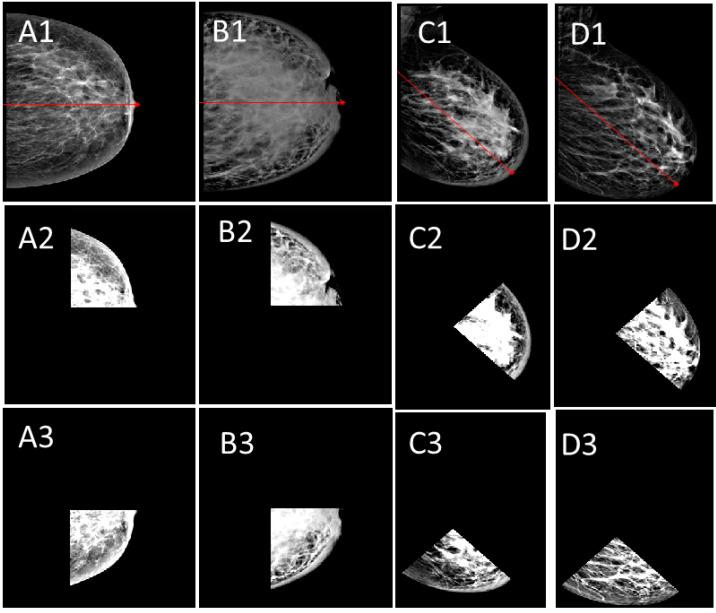
Identification of the entire breast, the view of the image as MLO or CC, and nipple location. The nipple location algorithm runs based on the view of the mammogram. The MLO and CC views, their nipple location, and data augmentation are shown. The red line is placed by an automatic nipple location algorithm, it divides the breast in half as the nipple location being the reference point. (**A1**–**A3**,**B1**–**B3**) show the segmentation of the quarters from CC views while (**C1**–**C3**,**D1**–**D3**) show the segmented quarters from MLO views.

Preprocessing stage implements artifact removal, breast localization, and orientation detection as CC or MLO, linear contrast stretch following a decorrelation stretch, and data augmentation. Figure 2a–d shows the resulting images of the preprocessing stage. The nipple location is determined as a reference point for the data augmentation and feature extraction. 

Decorrelation stretch is a linear pixel-based operation. It enhances the intensity differences. This process is performed after the artifact removal. 

### 3.3. Feature Extraction 

Mass, skin thickening, and nipple retraction are analyzed by size, texture, shape, and location. IBC cases may share similar characteristics with non-IBC and granulomatous mastitis cases, adding complexity to IBC detection and diagnosis. The following features were identified after extensive discussions with the team members, who are epidemiologists, radiologists, and oncologists. Breast thickness measured during mammographic imaging can also disclose information about IBC since the compression force can vary due to the pain from related breast inflammation. 

Mass:

- The density of a mass relative to its surrounding tissues: hypodense, isodense, hyperdense.
- Mass boundary shape: round, oval, irregular, macrolobulated, microlobulated, speculated.
- Mass texture: homogeneous, heterogeneous, fatty.
- Mass edge: sharp (well-circumscribed), ill-defined (fuzzy).
- Signs of symmetry, bilateral asymmetry.
- Location of the mass: entire, close to the nipple, above/below the nipple.
- Mass size.
- Number of masses.
- The ratio of the breast density of both breasts.

Skin thickness:

- Present only around the nipple.
- Present for the whole breast.
- Measurement of the skin thickness represented as the area.
- The percentage of the skin thickness of both breasts.

Nipple retraction:

- The level (degree) of nipple retraction.
- Nipple-mass connectivity.

As proof of concept, we analyzed the major IBC characteristics defined by AJCC criteria. These are (1) breast skin thickening, (2) its percentage, (3) the level of nipple retraction, (4) bilateral breast density asymmetry, and (5) the ratio of the breast density of both breasts in bilateral digital mammogram images. The ground truth (human diagnosis) was done by radiologists who are experts in IBC diagnosis.

**Figure 3:**
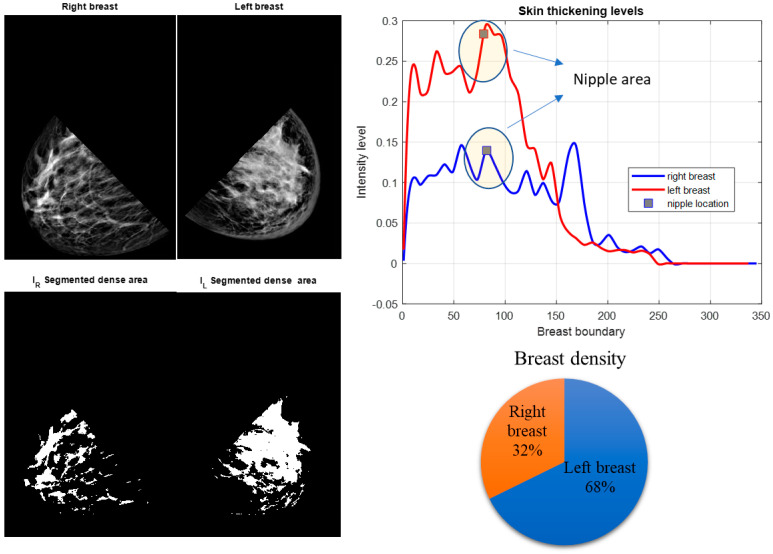
IBC patient. MLO view. The lower quadrants of the right and left breast are segmented. Breast density in both breasts is measured. The right and left breast skin thickening and density are calculated as average intensity values and compared. The left breast is clinically diagnosed with IBC while the right breast is normal. Figures show higher skin thickening and breast density for the left breast.

**Figure 4:**
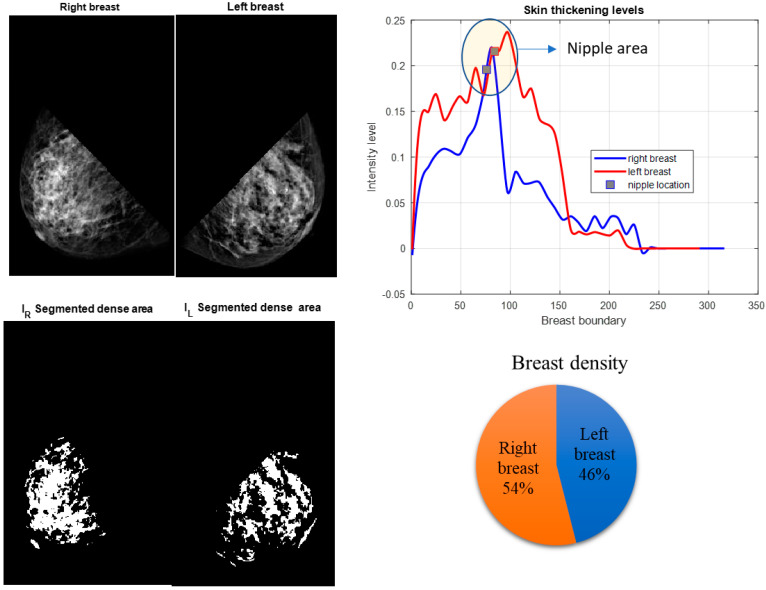
Non-IBC breast cancer patient. MLO view. The lower quadrants of the right and left breast are segmented. Breast density in both breasts is measured. The right and left breast skin thickening and density are calculated as average intensity values and compared. Both breasts showed comparable breast thickening and density measurements.

**Figure 5:**
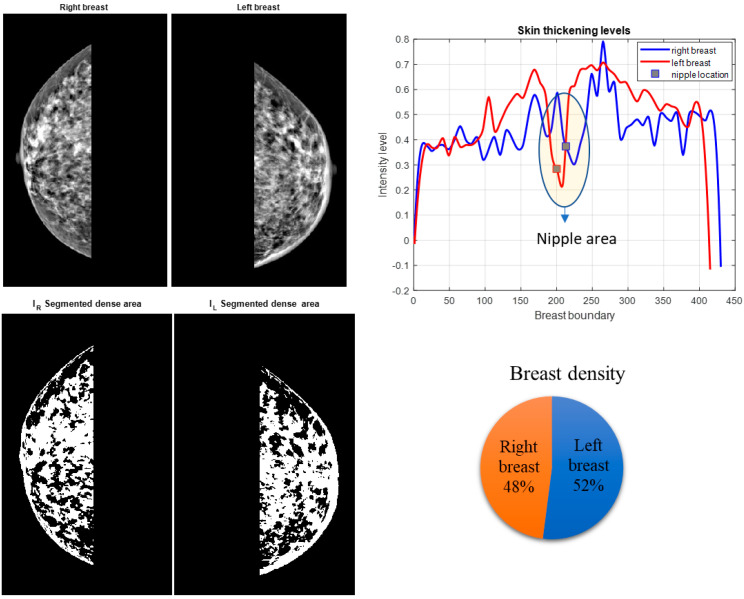
Benign case. CC view. The lower quadrants of the right and left breast are segmented. Breast density in both breasts is measured. The right and left breast skin thickening and density are calculated as average intensity values and compared. Both breasts showed comparable breast thickening and density measurements.

Our experimental results showed that digital mammography could successfully parameterize these features. Furthermore, results from the CC and MLO views of digital mammograms showed that the density ratio of right and left breasts could be another proficient feature in detecting IBC from mammograms. Some of the results of the proposed system are shown in Figure 3, Figure 4 and Figure 5. After segmenting the lower quadrants of the breasts, density and skin thickening are measured and compared.

We calculated two moments of IBC and non-IBC breast cancer cases as a preliminary analysis. These features are based on the mean value and standard deviation of the pixel intensities at the breast arc length of the right and left breasts, $\mu_{right}$, $\mu_{left}$, $\sigma_{right}$, and $\;\sigma_{left}$. The $I_{st}\left(x,y\right)$ is the segmented skin thickening region from a mammogram image and $g\left(z\right)$ denotes a function defined over the values of $\;z\epsilon \left[0,255\right]$ for the segmented region. The expected value of $g\left(z\right)$ can be defined as:

(1) $$E\left[g\left(z\right)\right]=\sum_{z\in \left[0,255\right]}g\left(z\right)p\left(z\right)$$

where $\;p\left(z\right)$ is the probability density function. The mean value, μ, can be calculated as:

(2) $$\mu =E\left[z\right]=\sum_{z\in \left[0,255\right]}z\;p\left(z\right)$$

Although mean value is an important statistical descriptor, we need to quantify the spreading of values about the mean, which will be the variance measurement:

(3) $$\sigma^{2}=E\left[{\left(z-\mu \right)}^{2}\right]=\sum_{z\epsilon \left[0,255\right]}{\left(z-\mu \right)}^{2}p\left(z\right)$$

In this work, we used the standard deviation, σ, which is the square root of the variance. Since standard deviation is proportional to the intensity and in a range comparable to the intensity values, it makes it easier to interpret skin thickening.

The mean values of the segmented breast density of the right and left breasts, $\gamma_{right}$ and $\;\gamma_{left}$, are calculated from the binary images shown in Figure 3b, Figure 4b and Figure 5b. Binary images are found by using Otsu’s thresholding method [28]. The mean value of the density is calculated for both breasts.

The MLO view of a bilateral digital mammogram of an IBC case is shown in Figure 3. The left breast displays skin thickening, one of the prominent markers of IBC. Skin thickening of both breasts is measured in the lower quadrants, as Figure 3 shows the left breast has higher skin thickening, which implies that the left breast may have IBC. Increased breast density is stated as one of the other characteristics of IBC. The segmented breast density for the lower quadrants of both breasts states that the left breast is denser than the right breast. This feature points to a possible IBC case accompanied by thicker skin on the left breast.

Table 1 shows the calculated moments and proposed features. 

The features are the ratios of the right breast to the left breast: (1) $\mu_{right}/\mu_{left}$ the ratio of the mean value of the pixel intensities at the skin thickening region, (2) $\sigma_{right}/\sigma_{left}\;$ the ratio of the standard deviations of the intensity values at the skin thickening region, and (3) $\gamma_{right}/\gamma_{left}$ the ratio of the mean values of the segmented breast densities.

**Figure 6:**
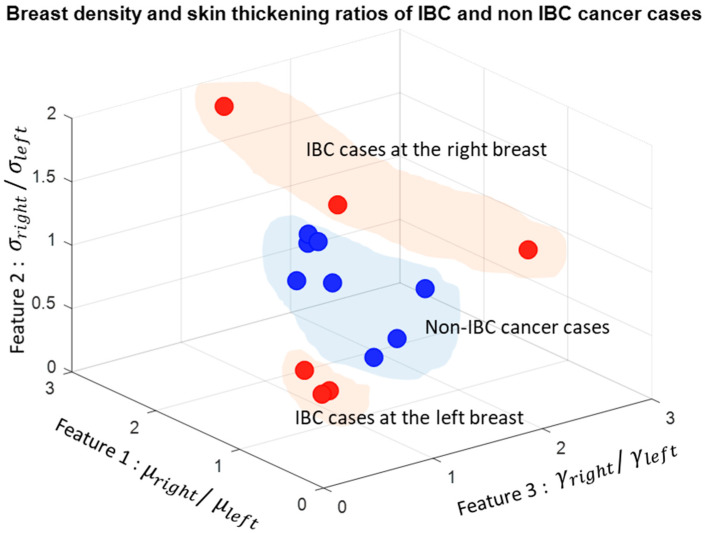
Three-dimensional (3D) illustration of the three features calculated from digital bilateral mammograms for IBC and non-IBC cancer cases. This figure is best viewed in color.

A 3D figure of these three features is plotted in Figure 6. For non-IBC breast cancer cases, these features have values around 1.0, while IBC cancer cases have lower or higher feature values depending on which breast has IBC. For instance, if the right breast has IBC, these features are calculated as greater than 1.0; if the left breast has IBC, these features are calculated as less than 1.0. 

After these features are extracted from the input mammogram image, the next step is to use these features as input to the CAD model to decide if IBC presents. This paper uses the fuzzy logic decision system to build the CAD model because of a small dataset. A detailed description of the CAD model is explained in the next section.

## 4. The Fuzzy Logic CAD Model

Machine learning algorithms, such as deep learning (DL), support vector machines (SVMs); artificial neural networks (ANNs) are empirical and their specifications are adjusted after experimentation. These algorithms are supervised and require large training datasets, especially for complex problems [28,29]. As IBC is rare, obtaining large training sets is challenging. Furthermore, rule-based systems do not require a training dataset. System rules are defined a priori by users; the system then provides a decision based on these rules. Rules are defined by systematically analyzing available clinical cases and the related image region of interest (ROI) characteristics. While the accuracy rates of the rule-based systems are lower than that of supervised techniques, they are typically more robust since performance does not depend on specific training data. Here, we designed a fuzzy logic-based CAD system that defined the rules through extensive analysis of healthy, IBC, and non-IBC cancer cases. 

**Figure 7:**
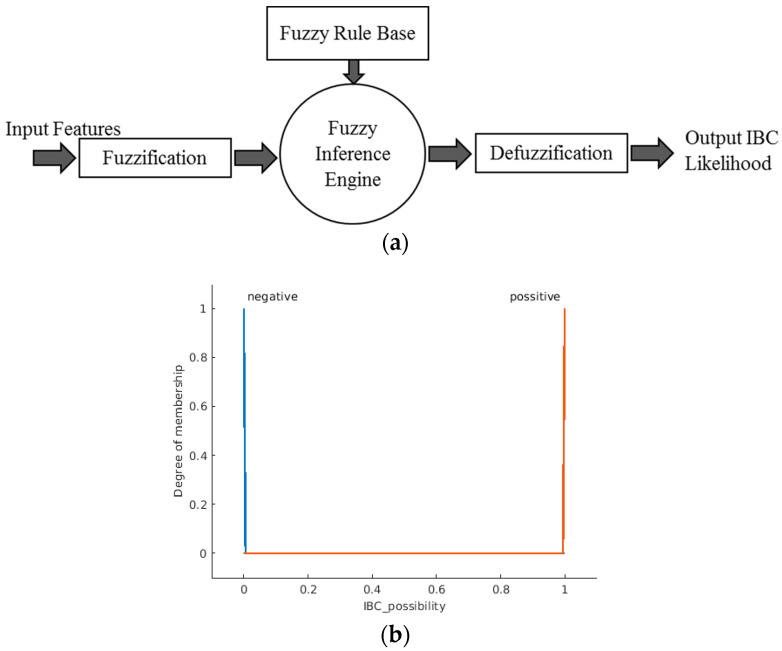
(**a**) The architecture of the proposed Type-1 fuzzy inference system. (**b**) Singleton output membership function used in the designed Type-1 fuzzy system.

**Figure 8:**
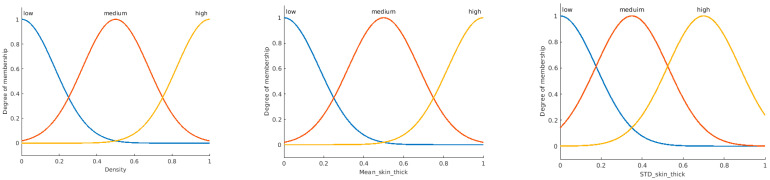
Three inputs proposed membership functions of the Type-1 fuzzy system.

The general structure of the Mamdani fuzzy inference system (FIS) is explained in Figure 7a [30,31]. The system consists of three main processes. The first process is fuzzification, where the input data is converted to fuzzy linguistic variables with a specific membership function. Figure 7b shows the proposed system’s output membership functions. The output membership functions are singleton functions to provide meaningful probabilistic classification crisp results. The final process is the defuzzification of the aggregated rules’ outputs to calculate the final crisp class probability. Figure 8 shows the proposed membership functions for the utilized three input variables. The second process is inference, where the user’s predefined rules are applied to the fuzzy input variables to predict the impact and weights of the inputs on the expected output. The anticipated output of the system must be declared as a fuzzy linguistic variable with membership functions to apply the rules. Fuzzy rules are given in Table 2.

**Figure 9:**
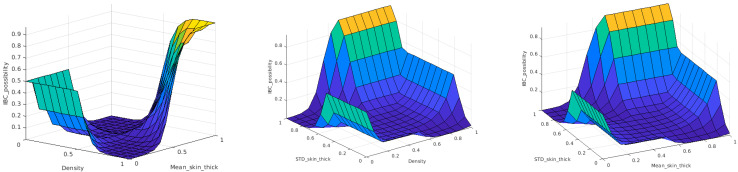
Decision surface of the proposed Type-1 fuzzy classifier.

The rule-based inference engine of the fuzzy CAD model implements the mapping function between input and output variables. An inference engine is a group of if–then rules that implements a fuzzy implication between input and output fuzzy linguistic variables. This function can be represented as a decision hypersurface. For visualization purposes, the decision surface can be represented between the output and two of the input variables at a time. Figure 9 shows the 3D view of the proposed decision hypersurface. The final defuzzyfied crisp output of the CAD system is considered as the likelihood of whether this patient is an IBC patient. A threshold of 0.5 is applied to these output possibilities to produce a binary output. If the output is greater than 0.5, the patient is considered an IBC case; otherwise, the patient will be regarded as a non-IBC case.

The Type-1 fuzzy system has only one level of membership function. That is why this structure is considered a Type-1 fuzzy system. For some cases, when the uncertainty is higher and the data amount is lower, defining more than one level of uncertainty is preferred to handle this ambiguity problem, as we face this problem in IBC diagnosis. This can be achieved by using a higher-order membership function for the Type-1 defined membership function. This newly defined membership function converts the system from a traditional Type-1 to a higher-order Type-2 fuzzy system. Several kinds of these Type-2 membership functions depend on the shape of this defined higher-order membership function [32]. 

**Figure 10:**
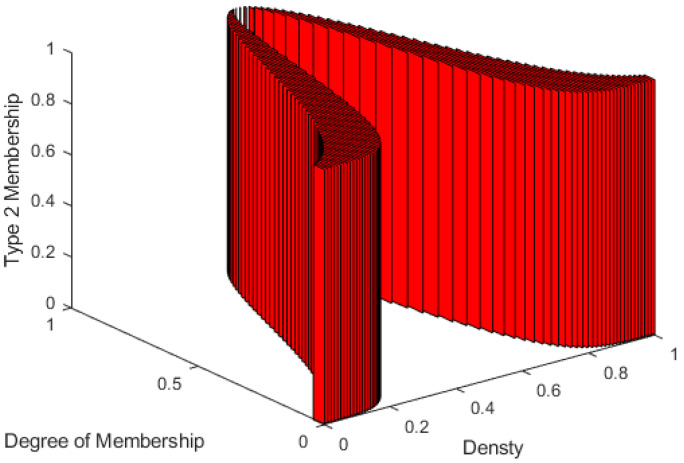
3D representation of medium Type-2 membership function used for the density input feature.

Figure 10 shows a 3D view of a uniform second-order membership function that was used in our work. This Type-2 fuzzy system is called Interval Type-2 Fuzzy System [33,34]. For each membership function in the Type-2 system, there are lower and upper-limit memberships. The distribution between the lower and upper limits of the defined membership function is called Footprint of Uncertainty (FoU), which is considered a uniform distribution in the Interval Type 2 Fuzzy Systems, as shown in Figure 10.

**Figure 11:**
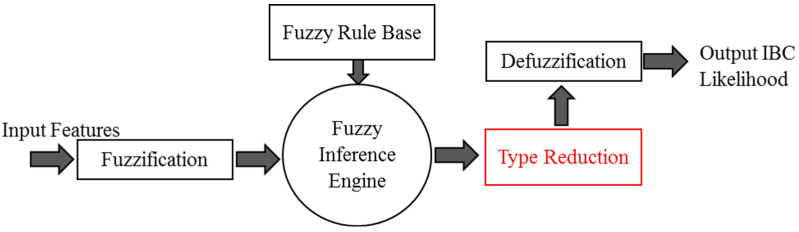
The architecture of the proposed Type-2 fuzzy inference system.

**Figure 12:**
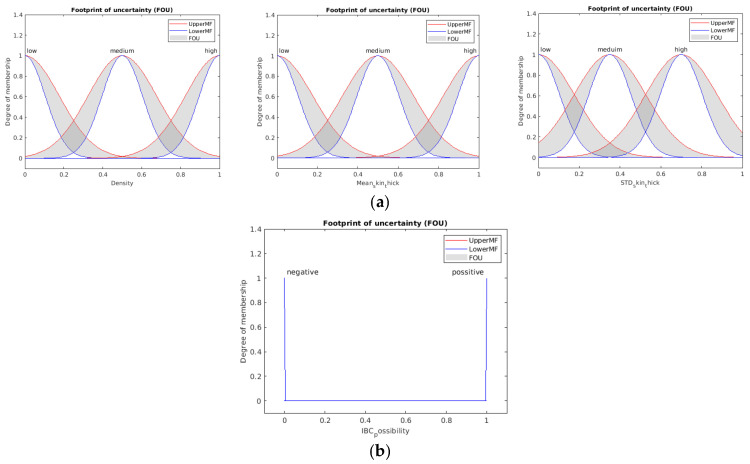
(**a**) Three inputs proposed membership functions of Type-1 fuzzy system. (**b**) Output membership function used in the designed Type-2 fuzzy system.

The general structure of the Type-2 fuzzy system is explained in Figure 11. The order reduction block is the main difference between Type-1 and Type-2 fuzzy systems. During the order reduction process, the output-implicated Type-2 membership function is reduced to a Type-1 membership function to apply the defuzzification technique and produce the required crisp possibilities likelihood output. Figure 12 shows the 2D representation of all inputs and output Type-2 fuzzy membership function utilized in the proposed system. As shown in Figure 12b, it is clear that because the output membership functions are singleton functions, they are similar for both Type-1 and Type-2 systems.

**Figure 13:**
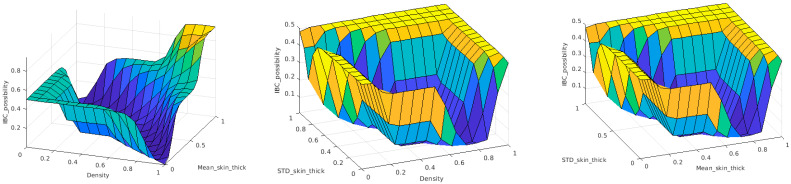
Decision surface of the proposed Type-2 fuzzy classifier.

The Type-2 system also implements fuzzy if–then rules that represent a function between input and output linguistic variables. Figure 13 shows the designed decision surfaces of the Type-2 fuzzy classifier. The output of the defuzzification process of the Type-2 classifier is also the possibility likelihood that the input mammogram features are for an IBC case or not. As in the Type-1 case, the output possibilities are thresholded with 0.5 to produce a binary decision as an IBC or a non-IBC case.

## 5. Results

The CAD output is compared to their corresponding ground truth data, the collaborating clinicians’ diagnosis. The key performance metrics of the CAD system are the true positive rate (an IBC case is correctly detected), true negative rate (probability that a non-IBC is correctly detected), FP is false positive (a non-IBC is detected as an IBC), and FN is the false negative (an IBC is detected as a non-IBC). Table 3 shows the calculated performance metrics of the designed classifier. From these measures, we compute the accuracy (ACC), recall, precision, area under the curve (AUC), and F1-score.

In addition to these measurements, the receiver operating characteristic (ROC) curve [35], an accepted standard in the machine learning field, will be derived to measure the performance of our CAD system. The ROC curve plots true positive ratios versus false positive ratios by varying the threshold on the probability map. The true-positive and false-positive ratios are defined as sensitivity and specificity, respectively. The value of the area under the ROC curve (AUC) is 1.0 for a perfect system.

The average computation time for feature extraction is five seconds per bilateral mammogram image. Type 1 and Type 2 fuzzy classifiers have one millisecond execution time per case. The overall CADx computation time is calculated at around five seconds. The system is designed to run as a cloud service that can dynamically allocate system resources depending on the required computation time to guarantee real-time performance.

## 6. Discussions and Conclusions

A CAD system creates a model that produces decisions using input data. There are ongoing research efforts to develop CAD systems in healthcare [36]. Machine learning algorithms allow researchers to design supervised models, but those models require large datasets for the training stage. Common diseases or frequently occurring health problems have sufficient datasets to develop ML-based CAD systems. However, inflammatory breast cancer (IBC) is a rare type of breast cancer, as medical experts and databases are scarce. Its roughly 55% 5-year survival rate makes IBC one of the most aggressive breast cancer types and increases the importance of early diagnosis. Radiologists and oncologists commonly use pathological findings, clinical images, mammography, ultrasound, and MRI images in IBC diagnosis. Limitations in IBC diagnosis occur when (1) there is no expert medical workforce in IBC diagnosis, (2) there is no access to imaging technology or labs, and (3) there is no access to advanced medical institutions and experts.

This work developed the first CADx system, a pioneering study in IBC diagnosis in the literature, and presented the proof of concept using bilateral digital mammography images. We used mammography images in the design since most medical centers worldwide are equipped with mammography systems. Instead of ML algorithms, the proposed fuzzy-based CADx has been designed based on transfer learning from human expertise to the fuzzy classifier, which does not require a training stage and does not require a large dataset. The medical team of IBC experts identified a list of IBC features in bilateral mammograms, and we focused on the skin thickness and higher density among those in this paper. Similar to IBC, cellulitis also exhibits skin thickening and subcutaneous edema in radiological imaging. Subcutaneous edema manifests as a diffuse increase in breast density [37]. The similar symptomology in these breast conditions makes detecting IBC in digital mammography images difficult. As the preliminary study, we did not include cellulitis cases in this work, only IBC and non-IBC cancer cases. Future work will add cellulitis cases to the image dataset to improve the CADx system. One note is that the proposed CADx model uses bilateral mammogram images in diagnosing IBC.

Although many believe that CAD systems could assist medical experts and improve the sensitivity in the diagnosis, others argue that increasing the recall rate due to using CAD might offset the potential benefit of a slight sensitivity increase. Hayat [38] stated that comparing performance differences based on true positives versus false positives generated by two observers, with or without using CAD, is difficult. It is difficult to determine which pair represents higher performance. The best approach to compensate for the variability of observer preference in choosing different operating thresholds of detection is to use ROC analysis. We presented the ROC curves of the designed Type 1 and 2 fuzzy logic classifiers. Type 2 fuzzy-based CADx achieved a perfect ROC curve.

This work extracted and analyzed breast skin thickening and its percentage, nipple retraction, breast density asymmetry, and the ratio of the breast density in bilateral mammograms. These features were used as inputs to Type 1 and 2 fuzzy interference systems. The results proved that we could successfully parameterize the corresponding IBC characteristics in mammograms. Future work will focus on acquiring more digital bilateral mammography images of IBC cases, extracting the other identified IBC characteristics here, and designing a Deep Neural Network (DNN)-based CADx model.

## Figures and Tables

**Table 1:** Skin thickening and breast density ratios of IBC and non-IBC cancer cases, including MLO and CC views. RB: Right breast, LB: Left breast.

Case Type and Number	Skin Thickening				Feature 1	Feature 2	Feature 3	Clinical Diagnosis
	$\mu_{right}$	$\mu_{left}$	$\sigma_{right}$	$\sigma_{left}$	$\mu_{right}/\mu_{left}$	$\sigma_{right}/\sigma_{left}$	$\gamma_{right}/\gamma_{left}$	IBC Diagnosis/Breast with IBC
IBC_1	0.06	0.10	0.05	0.11	0.55	0.48	0.48	Yes/LB
IBC_2	0.36	0.43	0.12	0.12	0.83	1.03	2.51	Yes/RB
IBC_3	0.24	0.40	0.09	0.14	0.61	0.67	0.30	Yes/LB
IBC_4	0.06	0.12	0.06	0.13	0.50	0.49	0.38	Yes/LB
IBC_5	0.36	0.17	0.10	0.09	2.14	1.16	1.78	Yes/RB
IBC_6	0.22	0.08	0.16	0.08	2.61	1.95	1.10	Yes/RB
NON-IBC_1	0.28	0.25	0.06	0.06	1.18	1.05	0.95	No
NON-IBC_2	0.17	0.15	0.12	0.11	1.12	1.14	0.62	No
NON-IBC_3	0.14	0.14	0.07	0.05	1.01	1.46	0.73	No
NON-IBC_4	0.07	0.11	0.05	0.08	0.62	0.61	0.94	No
NON-IBC_5	0.28	0.26	0.11	0.07	1.10	1.50	0.71	No
NON-IBC_6	0.14	0.12	0.12	0.09	1.15	1.40	0.75	No
NON-IBC_7	0.23	0.27	0.08	0.08	0.88	0.92	1.61	No
NON-IBC_8	0.06	0.09	0.06	0.08	0.65	0.70	1.17	No

**Table 2:** Fuzzy rules in the proposed Type-1 and Type-2 classifiers.

		Density								
		L			M			H		
		Mean								
		L	M	H	L	M	H	L	M	H
StandardDeviation	L	IBC	-	-	-	-	-	-	-	-
	M	-	-	-	-	NonIBC	-	-	-	-
	H	-	-	-	-	-	-	-	-	IBC

**Table 3:** Calculated performance metrics (%) of the designed fuzzy classifiers.

Method	Accuracy	Recall	Precision	AUC	F1-Score	Performance Metrics
Type-1 fuzzy CAD model	92.31	83.33	100	97.61	90.9	$Accuracy=\frac{TP+TN}{TP+TN+FP+FN}$ $Recall=\frac{TP}{TP+FN}$ $Precision=\frac{TP}{TP+FP}$ $F1\;score=\frac{2TP}{2TP+FP+FN}$
Type-2 fuzzy CAD model	100	100	100	100	100	

## Data Availability

Images and full medical reports can be found at https://wiki.cancerimagingarchive.net/pages/viewpage.action?pageId=109379611 (accessed on 28 October 2022).

## References

[1] Hance K.W., Anderson W.F., Devesa S.S., Young H.A., Levine P.H. (2005). Trends in inflammatory breast carcinoma incidence and survival: The surveillance, epidemiology, and end results program at the National Cancer Institute. JNCI J. Natl. Cancer Inst..

[2] Van Uden D.J.P., van Maaren M.C., Strobbe L.J.A., Bult P., van der Hoeven J.J., Siesling S., de Wilt J.H.W., Blanken-Peeters C.F.J.M. (2019). Metastatic behavior and overall survival according to breast cancer subtypes in stage IV inflammatory breast cancer. Breast Cancer Res..

[3] Rueth N.M., Lin H.Y., Bedrosian I., Shaitelman S.F., Ueno N.T., Shen Y., Babiera G. (2014). Underuse of trimodality treatment affects survival for patients with inflammatory breast cancer: An analysis of treatment and survival trends from the National Cancer Database. J. Clin. Oncol..

[4] Matsuda N., Wang X., Lim B., Krishnamurthy S., Alvarez R.H., Willey J.S., Parker C.A., Song J., Shen Y., Hu J. (2018). Safety and Efficacy of Panitumumab Plus Neoadjuvant Chemotherapy in Patients with Primary HER2-Negative Inflammatory Breast Cancer. JAMA Oncol..

[5] Boussen H., Bouzaiene H., Ben Hassouna J., Gamoudi A., Benna F., Rahal K. (2008). Inflammatory Breast Cancer in Tunisia: Reassessment of Incidence and Clinicopathological Features. Semin Oncol..

[6] Slaoui M., Zoure A.A., Mouh F.Z., Bensouda Y., El Mzibri M., Bakri Y., Amrani M. (2018). Outcome of Inflammatory Breast Cancer in Moroccan Patients: Clinical, Molecular and Pathological Characteristics of 219 Cases from the National Oncology Institute (INO). BMC Cancer.

[7] Soliman A.S., Kleer C.G., Mrad K., Karkouri M., Omar S., Khaled H.M., Benider A.L., Ayed F.B., Eissa S.S., Eissa M.S. (2011). Inflammatory Breast Cancer in North Africa: Comparison of Clinical and Molecular Epidemiologic Characteristics of Patients from Egypt, Tunisia, and Morocco. Breast Dis..

[8] Ueno N.T., Espinosa Fernandez J.R., Cristofanilli M., Overmoyer B., Rea D., Berdichevski F., El-Shinawi M., Bellon J., Le-Petross H.T., Lucci A. (2018). International Consensus on the Clinical Management of Inflammatory Breast Cancer from the Morgan Welch Inflammatory Breast Cancer Research Program 10th Anniversary Conference. J. Cancer.

[9] Schairer C., Hablas A., Seif Eldein I.A., Gaafar R., Rais H., Mezlini A., Ayed F.B., Ayoub W.B., Benider A., Tahri A. (2019). Clinicopathologic and Mammographic Characteristics of Inflammatory and Non-Inflammatory Breast Cancer at Six Centers in North Africa. Breast Cancer Res. Treat..

[10] Cristofanilli M., Buzdar A.U., Hortobágyi G.N. (2003). Update on the management of inflammatory breast cancer. Oncologist.

[11] Boutet G. (2012). Breast inflammation: Clinical examination, aetiological pointers. Diagn. Interv. Imaging.

[12] Schairer C., Soliman A.S., Omar S., Khaled H., Eissa S., Ayed F.B., Khalafallah S., Ayoub W.B., Kantor E.D., Merajver S. (2013). Assessment of Diagnosis of Inflammatory Breast Cancer Cases at Two Cancer Centers in Egypt and Tunisia. Cancer Med..

[13] Schairer C., Hablas A., Eldein I.A.S., Gaafar R., Rais H., Mezlini A., Ayed F.B., Ayoub W.B., Benider A., Tahri A. (2020). Risk Factors for Inflammatory and Non-Inflammatory Breast Cancer in North Africa. Breast Cancer Res. Treat..

[14] Yang W.T. (2010). Advances in Imaging of Inflammatory Breast Cancer. Cancer.

[15] Jagsi R., Mason G., Overmoyer B.A., Woodward W.A., Badve S., Schneider R.J., Lang J.E., Alpaugh M., Williams K.P., Vaught D. (2022). Inflammatory breast cancer defined: Proposed common diagnostic criteria to guide treatment and research. Breast Cancer Res. Treat..

[16] Yang W.T., Le-Petross H.T., Macapinlac H., Carkaci S., Gonzalez-Angulo A.M., Dawood S., Resetkova E., Hortobagyi G.N., Cristofanilli M. (2008). Inflammatory breast cancer: PET/CT, MRI, mammography, and sonography findings. Breast Cancer Res. Treat..

[17] Günhan-Bilgen I., Üstün E.E., Memiş A. (2002). Inflammatory Breast Carcinoma: Mammographic, Ultrasonographic, Clinical, and Pathologic Findings in 142 Cases. Radiology.

[18] Alunni J.-P. (2012). Imagerie du cancer du sein inflammatoire. J. Radiol. Diagn. Interv..

[19] Papalouka V., Gilbert F.J. (2018). Inflammatory Breast Cancer-Importance of Breast Imaging. Eur. J. Surg. Oncol..

[20] Lee K.W., Chung S.Y., Yang I., Kim H.D., Shin S.J., Kim J.E., Chung B.W., Choi J.A. (2005). Inflammatory Breast Cancer: Imaging Findings. Clin. Imaging.

[21] Le-Petross C.H., Bidaut L., Yang W.T. (2008). Evolving Role of Imaging Modalities in Inflammatory Breast Cancer. Semin Oncol..

[22] Renz D.M., Baltzer P.A., Böttcher J., Thaher F., Gajda M., Camara O., Runnebaum I.B., Kaiser W.A. (2008). Inflammatory Breast Carcinoma in Magnetic Resonance Imaging: A Comparison with Locally Advanced Breast Cancer. Acad. Radiol..

[23] Leong P.W., Chotai N.C., Kulkarni S. (2018). Imaging Features of Inflammatory Breast Disorders: A Pictorial Essay. Korean J. Radiol..

[24] Vertakova Krakovska B., Vanovcanova L. (2021). Inflammatory Breast Cancer: What Information We Can Expect from MRI and How to Use It. Ann. Oncol..

[25] Mamouch F., Berrada N., Aoullay Z., El Khanoussi B., Errihani H. (2018). Inflammatory Breast Cancer: A Literature Review. World J. Oncol..

[26] Yaghoobi R., Talaizade A., Lal K., Ranjbari N., Sohrabiaan N., Feily A. (2015). Inflammatory Breast Carcinoma Presenting with Two Different Patterns of Cutaneous Metastases: Carcinoma Telangiectaticum and Carcinoma Erysipeloides. J. Clin. Aesthet. Dermatol..

[27] Cancer Imaging Archive, Categorized Digital Database for Low energy and Subtracted Contrast Enhanced Spectral Mammography images (CDD-CESM). https://wiki.cancerimagingarchive.net/pages/viewpage.action?pageId=109379611.

[28] Barkana B.D., Saricicek I. (2017). Classification of Breast Masses in Mammograms Using 2D Homomorphic Transform Features and Supervised Classifiers. J. Med. Imaging Health Inform..

[29] Abu Mallouh A., Qawaqneh Z., Barkana B.D. (2019). Utilizing CNNs and Transfer Learning of Pre-Trained Models for Age Range Classification from Unconstrained Face Images. Image Vis. Comput..

[30] Ross T.J. (2016). Fuzzy Logic with Engineering Applications.

[31] Mendel J.M. (1995). Fuzzy Logic Systems for Engineering: A Tutorial. Proc. IEEE.

[32] Mendel J.M. (2017). Uncertain Rule-Based Fuzzy Systems: Introduction and New Directions.

[33] Mendel J.M., John R.I., Liu F. (2006). Interval Type-2 Fuzzy Logic Systems Made Simple. IEEE Trans. Fuzzy Syst..

[34] Castillo O., Melin P. (2014). A Review on Interval Type-2 Fuzzy Logic Applications in Intelligent Control. Inf. Sci..

[35] Davis J., Goadrich M. (2006). The Relationship between Precision-Recall and ROC Curves. Proceedings of the 23rd International Conference on Machine learning.

[36] Madani M., Mahdi Behzadi M., Navabi S. (2022). The Role of Deep Learning in Advancing Breast Cancer Detection Using Different Imaging Modalities: A Systematic Review. Cancers.

[37] Le-Petross H.T., Cristofanilli M., Carkaci S., Krishnamurthy S., Jackson E.F., Harrell R.K., Reed B.J., Yang W.T. (2011). MRI Features of Inflammatory Breast Cancer. Am. J. Roentgenol..

[38] Hayat M.A. (2008). Methods of Cancer Diagnosis, Therapy, and Prognosis, Volume 1 Breast Carcinoma.

